# Poly[[diaqua­bis­(2,2′-bipyridine)­bis­(μ_3_-5-hy­droxy­isophthalato)(μ_2_-5-hy­droxy­isophthalato)digadolinium(III)] trihydrate]

**DOI:** 10.1107/S1600536811035999

**Published:** 2011-09-14

**Authors:** Yan-Lin Zhang

**Affiliations:** a School of Chemistry and the Environment, South China Normal University, Guangzhou 510006, People’s Republic of China

## Abstract

The asymmetric unit of the title coordination polymer, {[Gd_2_(C_8_H_4_O_5_)_3_(C_10_H_8_N_2_)_2_(H_2_O)_2_]·3H_2_O}_*n*_, contains two Gd^III^ cations, one of which is surrounded by three 5-hy­droxy­isophthalate anions, one 2,2′-bipyridine ligand and two water mol­ecules in a distorted N_2_O_7_ tricapped trigonal–prismatic coordination geometry. The other Gd cation is coordinated by four 5-hy­droxy­isophthalate anions and one 2,2′-bipyridine ligand in a distorted N_2_O_7_ tricapped trigonal–prismatic coordination geometry. The 5-hy­droxy­isophthalate anions bridge the Gd cations, forming a layer structure. The layers are further connected by extensive O—H⋯O hydrogen bonding, assembling a three-dimensional supra­molecular network.

## Related literature

For metal organic frameworks (MOFs) with porous structures, see: Kitagawa *et al.* (2004 ▶); Kitaura *et al.* (2003 ▶); Chen *et al.* (2006 ▶); Luo *et al.* (2004 ▶); Xu *et al.* (2007 ▶). For a series of highly porous MOFs with bifunctional 1,4-benzene­dicarboxyl­ate (BDC) or trifunctional 1,3,5-benzene­tricarboxyl­ate (BTC), see: Eddaoudi *et al.* (2002 ▶). For complexes of *d*-block transition metal and *f*-block lanthanide ions, see: Lee *et al.* (2005 ▶); Sun *et al.* (2005 ▶).
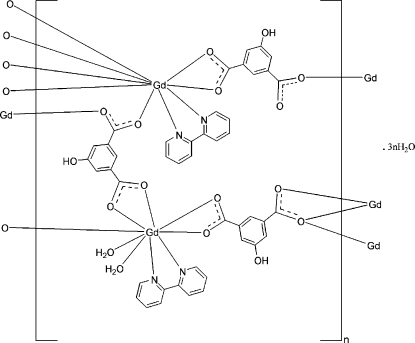


## Experimental

### 

#### Crystal data

[Gd_2_(C_8_H_4_O_5_)_3_(C_10_H_8_N_2_)_2_(H_2_O)_2_]·3H_2_O
*M* *_r_* = 1257.28
Triclinic, 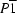

*a* = 11.4196 (13) Å
*b* = 12.0357 (14) Å
*c* = 17.886 (2) Å
α = 91.008 (1)°
β = 103.204 (1)°
γ = 106.648 (1)°
*V* = 2283.8 (5) Å^3^
*Z* = 2
Mo *K*α radiation
μ = 2.97 mm^−1^
*T* = 298 K
0.31 × 0.28 × 0.22 mm

#### Data collection

Bruker APEXII area-detector diffractometer
Absorption correction: multi-scan (*SADABS*; Bruker, 2001 ▶) *T*_min_ = 0.415, *T*_max_ = 0.521
11916 measured reflections
8089 independent reflections
6783 reflections with *I* > 2σ(*I*)
*R*_int_ = 0.023

#### Refinement

*R*[*F* ^2^ > 2σ(*F* ^2^)] = 0.032
*wR*(*F* ^2^) = 0.076
*S* = 1.05
8089 reflections
634 parameters
252 restraints
H-atom parameters constrained
Δρ_max_ = 1.17 e Å^−3^
Δρ_min_ = −1.33 e Å^−3^

### 

Data collection: *APEX2* (Bruker, 2007 ▶); cell refinement: *SAINT* (Bruker, 2007 ▶); data reduction: *SAINT*; program(s) used to solve structure: *SHELXS97* (Sheldrick, 2008 ▶); program(s) used to refine structure: *SHELXL97* (Sheldrick, 2008 ▶); molecular graphics: *ORTEPIII* (Burnett & Johnson, 1996 ▶); software used to prepare material for publication: *SHELXL97*.

## Supplementary Material

Crystal structure: contains datablock(s) I, global. DOI: 10.1107/S1600536811035999/xu5312sup1.cif

Structure factors: contains datablock(s) I. DOI: 10.1107/S1600536811035999/xu5312Isup2.hkl

Additional supplementary materials:  crystallographic information; 3D view; checkCIF report

## Figures and Tables

**Table 1:** Selected bond lengths (Å)

Gd1—O3	2.317 (3)
Gd1—O4^i^	2.385 (3)
Gd1—O6	2.473 (3)
Gd1—O7	2.503 (3)
Gd1—O13^ii^	2.383 (3)
Gd1—O14^ii^	2.769 (4)
Gd1—O14^iii^	2.360 (3)
Gd1—N3	2.624 (4)
Gd1—N4	2.568 (4)
Gd2—O1	2.539 (3)
Gd2—O2	2.426 (3)
Gd2—O8^iv^	2.334 (3)
Gd2—O11	2.496 (3)
Gd2—O12	2.488 (3)
Gd2—O1*W*	2.369 (4)
Gd2—O2*W*	2.463 (4)
Gd2—N1	2.570 (5)
Gd2—N2	2.651 (5)

Symmetry codes: (i) 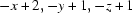; (ii) 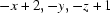; (iii) 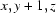; (iv) 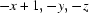.

**Table 2:** Hydrogen-bond geometry (Å, °)

*D*—H⋯*A*	*D*—H	H⋯*A*	*D*⋯*A*	*D*—H⋯*A*
O5—H5⋯O12^v^	0.82	1.92	2.720 (5)	164
O10—H10*A*⋯O11^vi^	0.82	1.96	2.755 (5)	163
O15—H15⋯O4*W*^vii^	0.82	2.13	2.942 (10)	171
O1*W*—H1*WA*⋯O3*W*	0.85	2.02	2.709 (6)	137
O1*W*—H1*WB*⋯O9^iv^	0.85	1.90	2.622 (5)	142
O2*W*—H2*WA*⋯O2	0.85	2.15	2.691 (5)	121
O2*W*—H2*WB*⋯O7^viii^	0.85	1.85	2.680 (5)	165
O3*W*—H3*WA*⋯O9	0.85	2.25	2.821 (7)	125
O3*W*—H3*WB*⋯O4*W*	0.85	2.19	2.713 (7)	119
O4*W*—H4*WA*⋯O6	0.85	1.97	2.813 (6)	171
O4*W*—H4*WB*⋯O1	0.85	2.18	2.797 (6)	129
O5*W*—H5*WA*⋯O15^iii^	0.85	1.97	2.817 (12)	173
O5*W*—H5*WB*⋯O10^ix^	0.85	2.19	3.006 (13)	160

Symmetry codes: (iii) 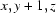; (iv) 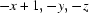; (v) 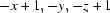; (vi) 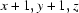; (vii) 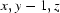; (viii) 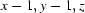; (ix) 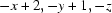.

**Fig. 1.**
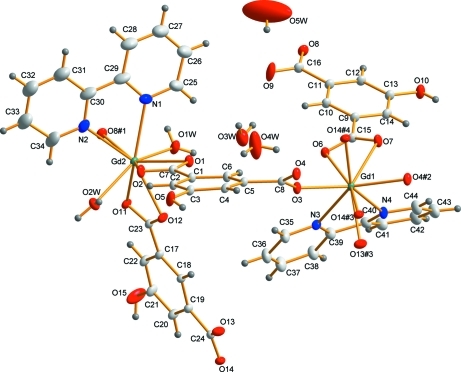
The asymmetric unit of the title compound, displacement ellipsoids drawn at the 30% probability level.

## supplementary crystallographic information

### Comment

In recent years, research on coordination polymers has made considerable progress in the fields (Luo *et al.*, 2004; Xu *et al.*, 2007). Especially, Over the past few decades considerable efforts have been placed on the synthesis of metal organic framework (MOF) with porous structures (Kitagawa *et al.*, 2004; Kitaura *et al.*, 2003). The rigid organic ring multidentate carboxylates have been generally used in this field (Chen *et al.*, 2006). Eddaoudi *et al.* succeeded in preparing a series of highly porous MOFs by bifunctional 1,4-benzenedicarb-oxylate (BDC) or trifunctional 1,3,5-benzenetricarboxylate (BTC) (Eddaoudi *et al.*, 2002). On the other hand, investigations of the phenyl-enedioxydiacetic acid complexes have mainly focused on the d-block transition-metal, f-block lanthanide ions have received comparatively less attention than transition-metal ions (Lee *et al.*, 2005). However, due to their ability of high coordination number, special magnetic and fluorescence properties, lanthanide complexes is likely to bring unprecedented crystal structures and unique properties (Sun *et al.*, 2005). So, new synthetic methods to obtain lanthanide coordination polymers with novel intrinsic porous still remain challenging. In this work, we synthesized successfully the new MOF with porous structure by using Gd rare earth metal, 5-Hydroxyisophthalate ligand and 2,2'-bipyridyl ligand under hydrothermal conditions.

The molecular structure the title compound is illustrated in Fig. 1, the asymmetric unit contains two Gd(III) ions, three 5-Hydroxyisophthalate ligands, two bipyridyl ligands and two water molecules. The Gd ion is nine-coordinated by five O atoms from three 5-Hydroxyisophthalate ligands, two N atoms from bipyridyl ligand and two water molecules, forming a distorted tricapped trigonal prismatic geometry. The Gd···O bond distances range from 2.316 (4) to 2.764 (4) Å, and the Gd···N bond lengths vary from 2.568 (6) to 2.651 (6) Å.

In the crystal structure, Each Gd metal centre is connected by the 5-hydroxyisophthalate ligands and the bipyridyl ligands to produce a layer. The intermolecular O—H···O hydrogenbonds interactions (Table 1), involving 5-hydroxyisophthalate ligands, bipyridyl ligands and water molecules, linking further the layers into a three-dimensional supramolecular network.

### Experimental

A mixture of Gd_2_O_3_ (0.363 g, 1 mmol), 5-hydroxyisophthalato acid (0.182 g, 1 mmol), 2,2'-bipyridine (0.132 g, 1 mmol), water (10 ml) in the presence of HClO_4_ (0.039 g, 0.385 mmol) was stirred vigorously for 30 min and then sealed in a Teflon-lined stainless-steel autoclave (20 ml capacity). The autoclave was heated and maintained at 433 K for 50 h, and then cooled to room temperature at 5 K.h^-1^ and obtained the colorless block crystals.

### Refinement

Water and hydroxy H atoms were tentatively located in difference Fourier maps and fixed in refinements, with distance restraints of O–H = 0.85 Å and H···H = 1.35 Å for water H atoms and o—H = 0.82 Å for hydroxy H atoms, *U*_iso_(H) = 1.5 *U*_eq_(O). Other H atoms were placed in calculated positions with C–H = 0.93 Å, and refined in riding mode with *U*_iso_(H) = 1.2*U*_eq_(C).

### Crystal data

**Table:**

[Gd_2_(C_8_H_4_O_5_)_3_(C_10_H_8_N_2_)_2_(H_2_O)_2_]·3H_2_O	*Z* = 2
*M**_r_* = 1257.28	*F*(000) = 1236
Triclinic, *P*1	*D*_x_ = 1.828 Mg m^−^^3^
Hall symbol: -P 1	Mo *K*α radiation, λ = 0.71073 Å
*a* = 11.4196 (13) Å	Cell parameters from 4955 reflections
*b* = 12.0357 (14) Å	θ = 2.4–25.2°
*c* = 17.886 (2) Å	µ = 2.97 mm^−^^1^
α = 91.008 (1)°	*T* = 298 K
β = 103.204 (1)°	Block, colourless
γ = 106.648 (1)°	0.31 × 0.28 × 0.22 mm
*V* = 2283.8 (5) Å^3^	

### Data collection

**Table:**

Bruker APEXII area-detector diffractometer	8089 independent reflections
Radiation source: fine-focus sealed tube	6783 reflections with *I* > 2σ(*I*)
graphite	*R*_int_ = 0.023
φ and ω scan	θ_max_ = 25.2°, θ_min_ = 1.8°
Absorption correction: multi-scan (*SADABS*; Bruker, 2001)	*h* = −11→13
*T*_min_ = 0.415, *T*_max_ = 0.521	*k* = −14→13
11916 measured reflections	*l* = −20→21

### Refinement

**Table:**

Refinement on *F*^2^	Primary atom site location: structure-invariant direct methods
Least-squares matrix: full	Secondary atom site location: difference Fourier map
*R*[*F*^2^ > 2σ(*F*^2^)] = 0.032	Hydrogen site location: inferred from neighbouring sites
*wR*(*F*^2^) = 0.076	H-atom parameters constrained
*S* = 1.05	*w* = 1/[σ^2^(*F*_o_^2^) + (0.0268*P*)^2^ + 4.4136*P*] where *P* = (*F*_o_^2^ + 2*F*_c_^2^)/3
8089 reflections	(Δ/σ)_max_ = 0.001
634 parameters	Δρ_max_ = 1.17 e Å^−^^3^
252 restraints	Δρ_min_ = −1.33 e Å^−^^3^

### Special details

**Table:**

Geometry. All esds (except the esd in the dihedral angle between two l.s. planes) are estimated using the full covariance matrix. The cell esds are taken into account individually in the estimation of esds in distances, angles and torsion angles; correlations between esds in cell parameters are only used when they are defined by crystal symmetry. An approximate (isotropic) treatment of cell esds is used for estimating esds involving l.s. planes.
Refinement. Refinement of F^2^ against ALL reflections. The weighted R-factor wR and goodness of fit S are based on F^2^, conventional R-factors R are based on F, with F set to zero for negative F^2^. The threshold expression of F^2^ > 2sigma(F^2^) is used only for calculating R-factors(gt) etc. and is not relevant to the choice of reflections for refinement. R-factors based on F^2^ are statistically about twice as large as those based on F, and R- factors based on ALL data will be even larger.

### Fractional atomic coordinates and isotropic or equivalent isotropic displacement parameters (Å^2^)

**Table:**

	*x*	*y*	*z*	*U*_iso_*/*U*_eq_	
C1	0.4825 (5)	0.0836 (4)	0.3988 (3)	0.0229 (8)	
C2	0.4122 (5)	0.0772 (4)	0.4536 (3)	0.0237 (9)	
H2	0.3348	0.0203	0.4466	0.028*	
C3	0.4574 (4)	0.1555 (4)	0.5186 (3)	0.0230 (9)	
C4	0.5716 (4)	0.2425 (4)	0.5289 (3)	0.0222 (9)	
H4	0.5995	0.2978	0.5711	0.027*	
C5	0.6437 (4)	0.2460 (4)	0.4755 (3)	0.0209 (8)	
C6	0.5988 (5)	0.1667 (4)	0.4108 (3)	0.0231 (9)	
H6	0.6473	0.1694	0.3752	0.028*	
C7	0.4287 (5)	0.0026 (4)	0.3266 (3)	0.0249 (10)	
C8	0.7700 (4)	0.3348 (4)	0.4872 (3)	0.0212 (9)	
C9	1.0118 (5)	0.3921 (4)	0.1825 (3)	0.0243 (8)	
C10	0.9050 (5)	0.3313 (4)	0.1278 (3)	0.0259 (9)	
H10	0.8312	0.2947	0.1424	0.031*	
C11	0.9081 (5)	0.3250 (4)	0.0505 (3)	0.0259 (9)	
C12	1.0187 (5)	0.3779 (4)	0.0290 (3)	0.0253 (9)	
H12	1.0210	0.3726	−0.0225	0.030*	
C13	1.1264 (5)	0.4389 (4)	0.0845 (3)	0.0258 (9)	
C14	1.1241 (5)	0.4465 (4)	0.1617 (3)	0.0265 (9)	
H14	1.1959	0.4871	0.1990	0.032*	
C15	1.0120 (5)	0.4014 (4)	0.2666 (3)	0.0253 (10)	
C16	0.7912 (5)	0.2616 (5)	−0.0098 (3)	0.0288 (11)	
C17	0.5752 (5)	−0.3885 (4)	0.2507 (3)	0.0236 (8)	
C18	0.6735 (4)	−0.3617 (4)	0.3163 (3)	0.0230 (9)	
H18	0.6876	−0.2962	0.3494	0.028*	
C19	0.7513 (5)	−0.4335 (4)	0.3326 (3)	0.0246 (8)	
C20	0.7287 (5)	−0.5311 (5)	0.2836 (3)	0.0306 (10)	
H20	0.7795	−0.5797	0.2952	0.037*	
C21	0.6317 (5)	−0.5578 (5)	0.2177 (3)	0.0332 (10)	
C22	0.5546 (5)	−0.4859 (5)	0.2015 (3)	0.0312 (10)	
H22	0.4888	−0.5034	0.1574	0.037*	
C23	0.4846 (5)	−0.3179 (4)	0.2360 (3)	0.0234 (10)	
C24	0.8531 (5)	−0.4071 (4)	0.4055 (3)	0.0247 (10)	
C25	0.3528 (7)	0.0870 (6)	0.1175 (4)	0.0615 (16)	
H25	0.4352	0.0835	0.1307	0.074*	
C26	0.3300 (8)	0.1788 (6)	0.0774 (4)	0.0685 (15)	
H26	0.3937	0.2330	0.0616	0.082*	
C27	0.2111 (8)	0.1865 (7)	0.0623 (5)	0.0706 (15)	
H27	0.1921	0.2473	0.0356	0.085*	
C28	0.1188 (8)	0.1057 (7)	0.0858 (4)	0.0654 (14)	
H28	0.0378	0.1127	0.0769	0.078*	
C29	0.1472 (7)	0.0119 (6)	0.1236 (4)	0.0558 (13)	
C30	0.0507 (7)	−0.0824 (7)	0.1467 (4)	0.0584 (13)	
C31	−0.0743 (7)	−0.0819 (8)	0.1332 (5)	0.0735 (14)	
H31	−0.0981	−0.0185	0.1134	0.088*	
C32	−0.1609 (8)	−0.1762 (8)	0.1496 (5)	0.0803 (15)	
H32	−0.2445	−0.1768	0.1413	0.096*	
C33	−0.1269 (7)	−0.2695 (9)	0.1779 (5)	0.0771 (16)	
H33	−0.1863	−0.3357	0.1869	0.093*	
C34	−0.0001 (6)	−0.2620 (8)	0.1928 (4)	0.0693 (17)	
H34	0.0251	−0.3241	0.2138	0.083*	
C35	0.9127 (5)	0.0884 (5)	0.4210 (4)	0.0418 (12)	
H35	0.8578	0.1117	0.4445	0.050*	
C36	0.8929 (6)	−0.0290 (5)	0.4063 (4)	0.0480 (12)	
H36	0.8266	−0.0834	0.4197	0.058*	
C37	0.9721 (6)	−0.0639 (5)	0.3717 (4)	0.0493 (12)	
H37	0.9609	−0.1427	0.3612	0.059*	
C38	1.0695 (6)	0.0190 (5)	0.3523 (4)	0.0420 (11)	
H38	1.1241	−0.0033	0.3280	0.050*	
C39	1.0849 (5)	0.1352 (5)	0.3693 (3)	0.0326 (10)	
C40	1.1930 (5)	0.2272 (5)	0.3551 (3)	0.0315 (10)	
C41	1.2673 (5)	0.2066 (5)	0.3085 (3)	0.0380 (10)	
H41	1.2436	0.1354	0.2794	0.046*	
C42	1.3754 (6)	0.2904 (5)	0.3048 (4)	0.0432 (11)	
H42	1.4249	0.2773	0.2729	0.052*	
C43	1.4093 (6)	0.3935 (5)	0.3490 (4)	0.0461 (11)	
H43	1.4856	0.4497	0.3512	0.055*	
C44	1.3265 (6)	0.4121 (5)	0.3904 (4)	0.0450 (13)	
H44	1.3466	0.4842	0.4177	0.054*	
Gd1	1.04200 (2)	0.394578 (19)	0.429399 (12)	0.01701 (7)	
Gd2	0.32468 (2)	−0.17119 (2)	0.199574 (13)	0.02017 (7)	
N1	0.2649 (5)	0.0030 (4)	0.1387 (3)	0.0415 (12)	
N2	0.0875 (4)	−0.1705 (5)	0.1784 (3)	0.0398 (12)	
N3	1.0066 (4)	0.1707 (4)	0.4034 (2)	0.0257 (9)	
N4	1.2204 (4)	0.3324 (4)	0.3931 (3)	0.0308 (10)	
O1	0.4872 (3)	0.0106 (3)	0.27407 (19)	0.0286 (8)	
O2	0.3229 (3)	−0.0732 (3)	0.31882 (19)	0.0303 (9)	
O3	0.8478 (3)	0.3098 (3)	0.4555 (2)	0.0272 (8)	
O4	0.7914 (3)	0.4286 (3)	0.52814 (19)	0.0269 (8)	
O5	0.3858 (3)	0.1410 (4)	0.5713 (2)	0.0381 (10)	
H5	0.4290	0.1759	0.6128	0.057*	
O6	0.9359 (3)	0.3244 (3)	0.29311 (18)	0.0254 (8)	
O7	1.0911 (3)	0.4849 (3)	0.31090 (18)	0.0316 (9)	
O8	0.7983 (3)	0.2599 (3)	−0.07886 (18)	0.0303 (8)	
O9	0.6932 (4)	0.2138 (5)	0.0118 (2)	0.0644 (15)	
O10	1.2346 (3)	0.4877 (3)	0.0613 (2)	0.0356 (9)	
H10A	1.2891	0.5270	0.0979	0.053*	
O11	0.3877 (3)	−0.3498 (3)	0.18007 (19)	0.0274 (8)	
O12	0.5040 (3)	−0.2276 (3)	0.27956 (19)	0.0286 (8)	
O13	0.8553 (4)	−0.3317 (3)	0.4550 (2)	0.0340 (9)	
O14	0.9326 (3)	−0.4648 (3)	0.4180 (2)	0.0287 (8)	
O15	0.6061 (5)	−0.6548 (4)	0.1695 (3)	0.0720 (17)	
H15	0.6355	−0.7026	0.1928	0.108*	
O1W	0.4679 (3)	−0.1230 (3)	0.1195 (2)	0.0405 (10)	
H1WA	0.5434	−0.1102	0.1158	0.061*	
H1WB	0.4230	−0.1193	0.0752	0.061*	
O2W	0.2359 (3)	−0.3061 (3)	0.2883 (2)	0.0354 (9)	
H2WA	0.2351	−0.2565	0.3225	0.053*	
H2WB	0.2019	−0.3734	0.3007	0.053*	
O3W	0.6877 (4)	0.0285 (4)	0.1066 (3)	0.0650 (14)	
H3WA	0.7145	0.0524	0.0674	0.097*	
H3WB	0.6482	0.0736	0.1183	0.097*	
O4W	0.7005 (6)	0.1565 (6)	0.2362 (3)	0.121 (3)	
H4WA	0.7746	0.2012	0.2552	0.181*	
H4WB	0.6638	0.1415	0.2728	0.181*	
O5W	0.5593 (16)	0.4214 (15)	0.0206 (8)	0.356 (12)	
H5WA	0.5768	0.4043	0.0669	0.533*	
H5WB	0.6263	0.4357	0.0046	0.533*	

### Atomic displacement parameters (Å^2^)

**Table:**

	*U*^11^	*U*^22^	*U*^33^	*U*^12^	*U*^13^	*U*^23^
C1	0.0213 (17)	0.0219 (17)	0.0218 (17)	0.0028 (14)	0.0031 (15)	−0.0031 (14)
C2	0.0192 (18)	0.0252 (18)	0.0216 (18)	0.0005 (16)	0.0028 (16)	−0.0028 (16)
C3	0.0189 (18)	0.0272 (18)	0.0214 (18)	0.0055 (16)	0.0041 (16)	−0.0038 (16)
C4	0.0191 (18)	0.0255 (18)	0.0202 (17)	0.0068 (15)	0.0019 (16)	−0.0052 (15)
C5	0.0187 (17)	0.0225 (16)	0.0196 (16)	0.0046 (14)	0.0031 (14)	−0.0024 (14)
C6	0.0207 (18)	0.0247 (18)	0.0218 (18)	0.0028 (15)	0.0066 (16)	−0.0032 (15)
C7	0.024 (2)	0.022 (2)	0.022 (2)	0.0029 (18)	−0.0009 (19)	−0.0022 (18)
C8	0.020 (2)	0.023 (2)	0.0171 (19)	0.0051 (17)	0.0015 (18)	−0.0016 (17)
C9	0.0251 (17)	0.0281 (17)	0.0161 (16)	0.0051 (15)	0.0014 (15)	−0.0013 (14)
C10	0.0256 (19)	0.0298 (19)	0.0169 (17)	0.0034 (16)	0.0010 (16)	−0.0008 (16)
C11	0.0268 (18)	0.0295 (17)	0.0181 (16)	0.0066 (15)	0.0011 (15)	−0.0015 (15)
C12	0.0271 (19)	0.0302 (19)	0.0155 (17)	0.0059 (17)	0.0025 (16)	−0.0017 (16)
C13	0.0255 (19)	0.0299 (19)	0.0190 (18)	0.0058 (17)	0.0028 (16)	−0.0017 (16)
C14	0.0250 (19)	0.0292 (19)	0.0199 (18)	0.0052 (17)	−0.0011 (16)	−0.0040 (16)
C15	0.025 (2)	0.027 (2)	0.019 (2)	0.0050 (19)	0.0008 (19)	−0.0006 (18)
C16	0.030 (2)	0.031 (2)	0.018 (2)	0.004 (2)	−0.0010 (19)	−0.0016 (18)
C17	0.0192 (17)	0.0282 (17)	0.0210 (16)	0.0065 (15)	0.0014 (15)	−0.0018 (15)
C18	0.0216 (19)	0.0234 (18)	0.0212 (18)	0.0060 (16)	0.0007 (16)	0.0003 (16)
C19	0.0223 (17)	0.0253 (17)	0.0230 (17)	0.0058 (15)	0.0009 (15)	0.0006 (15)
C20	0.027 (2)	0.032 (2)	0.0305 (19)	0.0115 (17)	−0.0015 (17)	−0.0056 (17)
C21	0.028 (2)	0.036 (2)	0.0306 (19)	0.0107 (17)	−0.0035 (17)	−0.0122 (17)
C22	0.0241 (19)	0.036 (2)	0.0279 (19)	0.0086 (17)	−0.0036 (17)	−0.0081 (17)
C23	0.019 (2)	0.026 (2)	0.021 (2)	0.0048 (18)	0.0004 (18)	0.0000 (18)
C24	0.022 (2)	0.022 (2)	0.025 (2)	0.0029 (18)	0.0014 (18)	0.0041 (18)
C25	0.064 (3)	0.040 (3)	0.066 (3)	0.011 (3)	−0.008 (3)	0.013 (3)
C26	0.073 (3)	0.046 (3)	0.071 (3)	0.013 (2)	−0.008 (3)	0.012 (2)
C27	0.076 (3)	0.055 (3)	0.074 (3)	0.026 (2)	−0.004 (3)	0.009 (2)
C28	0.068 (3)	0.064 (3)	0.068 (3)	0.038 (2)	0.001 (2)	0.006 (2)
C29	0.058 (3)	0.068 (3)	0.054 (3)	0.044 (2)	0.006 (2)	0.004 (2)
C30	0.051 (3)	0.084 (3)	0.055 (3)	0.044 (2)	0.012 (2)	0.009 (2)
C31	0.054 (3)	0.104 (3)	0.071 (3)	0.038 (2)	0.011 (2)	0.019 (3)
C32	0.049 (3)	0.117 (4)	0.078 (3)	0.030 (3)	0.014 (2)	0.023 (3)
C33	0.043 (3)	0.118 (4)	0.073 (3)	0.026 (3)	0.017 (3)	0.024 (3)
C34	0.036 (3)	0.113 (4)	0.064 (3)	0.025 (3)	0.017 (3)	0.023 (3)
C35	0.031 (3)	0.029 (2)	0.064 (3)	0.004 (2)	0.016 (2)	−0.001 (2)
C36	0.036 (2)	0.032 (2)	0.071 (3)	0.0003 (19)	0.017 (2)	−0.001 (2)
C37	0.043 (2)	0.032 (2)	0.069 (3)	0.0029 (19)	0.017 (2)	−0.005 (2)
C38	0.041 (2)	0.031 (2)	0.056 (2)	0.0072 (18)	0.019 (2)	−0.0047 (19)
C39	0.035 (2)	0.027 (2)	0.039 (2)	0.0099 (17)	0.0149 (18)	−0.0011 (18)
C40	0.035 (2)	0.030 (2)	0.036 (2)	0.0132 (17)	0.0168 (18)	0.0051 (17)
C41	0.042 (2)	0.036 (2)	0.045 (2)	0.0151 (18)	0.0230 (19)	0.0057 (19)
C42	0.043 (2)	0.042 (2)	0.054 (2)	0.0133 (19)	0.029 (2)	0.009 (2)
C43	0.042 (2)	0.041 (2)	0.061 (3)	0.009 (2)	0.027 (2)	0.010 (2)
C44	0.040 (3)	0.038 (3)	0.062 (3)	0.008 (2)	0.027 (2)	0.004 (2)
Gd1	0.01518 (13)	0.01894 (13)	0.01425 (12)	0.00264 (10)	0.00175 (10)	−0.00201 (9)
Gd2	0.01919 (14)	0.02123 (14)	0.01529 (13)	0.00330 (10)	−0.00138 (10)	−0.00330 (9)
N1	0.051 (3)	0.032 (3)	0.034 (3)	0.013 (2)	−0.005 (2)	0.001 (2)
N2	0.028 (3)	0.060 (3)	0.031 (3)	0.013 (2)	0.007 (2)	−0.002 (2)
N3	0.023 (2)	0.025 (2)	0.030 (2)	0.0084 (19)	0.0050 (19)	−0.0025 (18)
N4	0.023 (2)	0.027 (2)	0.041 (3)	0.0024 (19)	0.011 (2)	−0.001 (2)
O1	0.027 (2)	0.030 (2)	0.0231 (18)	−0.0003 (16)	0.0068 (16)	−0.0061 (15)
O2	0.027 (2)	0.0260 (19)	0.0271 (19)	−0.0056 (16)	0.0039 (16)	−0.0111 (15)
O3	0.0204 (19)	0.0258 (19)	0.034 (2)	0.0017 (15)	0.0115 (16)	−0.0053 (15)
O4	0.0231 (19)	0.0243 (19)	0.0291 (19)	0.0000 (15)	0.0081 (16)	−0.0109 (15)
O5	0.023 (2)	0.059 (3)	0.024 (2)	−0.0033 (19)	0.0100 (17)	−0.0098 (18)
O6	0.0246 (19)	0.0271 (19)	0.0175 (17)	−0.0003 (15)	0.0022 (15)	−0.0022 (14)
O7	0.032 (2)	0.032 (2)	0.0172 (18)	−0.0090 (17)	0.0027 (16)	−0.0063 (15)
O8	0.031 (2)	0.039 (2)	0.0160 (18)	0.0063 (17)	0.0012 (16)	−0.0043 (15)
O9	0.030 (2)	0.107 (4)	0.026 (2)	−0.021 (3)	0.0001 (19)	−0.007 (2)
O10	0.027 (2)	0.045 (2)	0.029 (2)	−0.0016 (18)	0.0107 (17)	−0.0085 (17)
O11	0.0230 (19)	0.030 (2)	0.0229 (18)	0.0077 (16)	−0.0058 (16)	−0.0059 (15)
O12	0.026 (2)	0.0266 (19)	0.0258 (19)	0.0078 (16)	−0.0072 (16)	−0.0083 (15)
O13	0.044 (2)	0.032 (2)	0.0222 (19)	0.0165 (18)	−0.0050 (17)	−0.0039 (16)
O14	0.026 (2)	0.030 (2)	0.030 (2)	0.0126 (16)	0.0024 (16)	0.0076 (16)
O15	0.079 (4)	0.066 (3)	0.061 (3)	0.045 (3)	−0.029 (3)	−0.044 (3)
O1W	0.026 (2)	0.058 (3)	0.027 (2)	−0.0005 (19)	0.0020 (17)	−0.0037 (18)
O2W	0.043 (2)	0.025 (2)	0.033 (2)	0.0017 (17)	0.0119 (19)	−0.0014 (16)
O3W	0.055 (3)	0.060 (3)	0.072 (3)	0.000 (2)	0.023 (3)	0.000 (3)
O4W	0.097 (5)	0.148 (6)	0.057 (4)	−0.065 (4)	0.033 (3)	−0.033 (4)
O5W	0.52 (3)	0.55 (3)	0.292 (16)	0.45 (2)	0.309 (18)	0.275 (18)

### Geometric parameters (Å, °)

**Table:**

C1—C6	1.383 (7)	C32—C33	1.359 (11)
C1—C2	1.393 (7)	C32—H32	0.9300
C1—C7	1.498 (7)	C33—C34	1.387 (10)
C2—C3	1.382 (6)	C33—H33	0.9300
C2—H2	0.9300	C34—N2	1.332 (9)
C3—O5	1.366 (6)	C34—H34	0.9300
C3—C4	1.391 (7)	C35—N3	1.337 (7)
C4—C5	1.391 (6)	C35—C36	1.376 (8)
C4—H4	0.9300	C35—H35	0.9300
C5—C6	1.387 (6)	C36—C37	1.356 (9)
C5—C8	1.495 (7)	C36—H36	0.9300
C6—H6	0.9300	C37—C38	1.381 (8)
C7—O1	1.261 (6)	C37—H37	0.9300
C7—O2	1.264 (6)	C38—C39	1.378 (7)
C8—O3	1.257 (6)	C38—H38	0.9300
C8—O4	1.266 (6)	C39—N3	1.346 (6)
C9—C10	1.378 (7)	C39—C40	1.478 (7)
C9—C14	1.398 (7)	C40—N4	1.344 (7)
C9—C15	1.506 (6)	C40—C41	1.383 (7)
C10—C11	1.392 (6)	C41—C42	1.368 (8)
C10—H10	0.9300	C41—H41	0.9300
C11—C12	1.386 (7)	C42—C43	1.365 (8)
C11—C16	1.503 (7)	C42—H42	0.9300
C12—C13	1.391 (7)	C43—C44	1.389 (8)
C12—H12	0.9300	C43—H43	0.9300
C13—O10	1.367 (6)	C44—N4	1.324 (7)
C13—C14	1.390 (7)	C44—H44	0.9300
C14—H14	0.9300	Gd1—O3	2.317 (3)
C15—O7	1.256 (6)	Gd1—O4^ii^	2.385 (3)
C15—O6	1.257 (6)	Gd1—O6	2.473 (3)
C16—O9	1.252 (6)	Gd1—O7	2.503 (3)
C16—O8	1.256 (6)	Gd1—O13^i^	2.383 (3)
C17—C22	1.383 (7)	Gd1—O14^i^	2.769 (4)
C17—C18	1.385 (6)	Gd1—O14^iii^	2.360 (3)
C17—C23	1.500 (7)	Gd1—N3	2.624 (4)
C18—C19	1.397 (7)	Gd1—N4	2.568 (4)
C18—H18	0.9300	Gd1—Gd1^ii^	4.0018 (5)
C19—C20	1.378 (7)	Gd2—O1	2.539 (3)
C19—C24	1.495 (7)	Gd2—O2	2.426 (3)
C20—C21	1.381 (7)	Gd2—O8^iv^	2.334 (3)
C20—H20	0.9300	Gd2—O11	2.496 (3)
C21—O15	1.358 (6)	Gd2—O12	2.488 (3)
C21—C22	1.392 (7)	Gd2—O1W	2.369 (4)
C22—H22	0.9300	Gd2—O2W	2.463 (4)
C23—O12	1.261 (6)	Gd2—N1	2.570 (5)
C23—O11	1.269 (6)	Gd2—N2	2.651 (5)
C24—O13	1.247 (6)	O5—H5	0.8200
C24—O14	1.277 (6)	O8—Gd2^iv^	2.334 (3)
C24—Gd1^i^	2.906 (5)	O10—H10A	0.8200
C25—N1	1.333 (8)	O13—Gd1^i^	2.383 (3)
C25—C26	1.382 (9)	O14—Gd1^v^	2.360 (3)
C25—H25	0.9300	O14—Gd1^i^	2.769 (4)
C26—C27	1.353 (11)	O15—H15	0.8200
C26—H26	0.9300	O1W—H1WA	0.8500
C27—C28	1.365 (10)	O1W—H1WB	0.8501
C27—H27	0.9300	O2W—H2WA	0.8500
C28—C29	1.405 (9)	O2W—H2WB	0.8500
C28—H28	0.9300	O3W—H3WA	0.8499
C29—N1	1.345 (8)	O3W—H3WB	0.8501
C29—C30	1.477 (10)	O4W—H4WA	0.8500
C30—N2	1.339 (8)	O4W—H4WB	0.8500
C30—C31	1.394 (9)	O5W—H5WA	0.8499
C31—C32	1.362 (11)	O5W—H5WB	0.8501
C31—H31	0.9300		
			
C6—C1—C2	119.5 (4)	N4—C44—C43	123.4 (6)
C6—C1—C7	121.2 (4)	N4—C44—H44	118.3
C2—C1—C7	119.3 (4)	C43—C44—H44	118.3
C3—C2—C1	120.0 (4)	O3—Gd1—O14^iii^	72.23 (12)
C3—C2—H2	120.0	O3—Gd1—O13^i^	90.37 (13)
C1—C2—H2	120.0	O14^iii^—Gd1—O13^i^	127.51 (12)
O5—C3—C2	116.7 (4)	O3—Gd1—O4^ii^	135.63 (11)
O5—C3—C4	122.7 (4)	O14^iii^—Gd1—O4^ii^	77.70 (12)
C2—C3—C4	120.5 (4)	O13^i^—Gd1—O4^ii^	82.72 (12)
C3—C4—C5	119.3 (4)	O3—Gd1—O6	86.22 (11)
C3—C4—H4	120.4	O14^iii^—Gd1—O6	88.21 (12)
C5—C4—H4	120.4	O13^i^—Gd1—O6	140.96 (12)
C6—C5—C4	120.0 (4)	O4^ii^—Gd1—O6	125.00 (11)
C6—C5—C8	119.5 (4)	O3—Gd1—O7	129.25 (12)
C4—C5—C8	120.5 (4)	O14^iii^—Gd1—O7	78.52 (12)
C1—C6—C5	120.5 (4)	O13^i^—Gd1—O7	139.80 (13)
C1—C6—H6	119.7	O4^ii^—Gd1—O7	73.20 (11)
C5—C6—H6	119.7	O6—Gd1—O7	51.86 (11)
O1—C7—O2	120.5 (4)	O3—Gd1—N4	138.95 (13)
O1—C7—C1	120.6 (4)	O14^iii^—Gd1—N4	145.72 (13)
O2—C7—C1	118.9 (4)	O13^i^—Gd1—N4	75.45 (14)
O3—C8—O4	124.7 (4)	O4^ii^—Gd1—N4	81.37 (12)
O3—C8—C5	116.9 (4)	O6—Gd1—N4	81.91 (13)
O4—C8—C5	118.4 (4)	O7—Gd1—N4	69.55 (14)
C10—C9—C14	121.0 (4)	O3—Gd1—N3	76.48 (12)
C10—C9—C15	121.2 (4)	O14^iii^—Gd1—N3	141.91 (12)
C14—C9—C15	117.8 (4)	O13^i^—Gd1—N3	73.01 (12)
C9—C10—C11	119.7 (5)	O4^ii^—Gd1—N3	140.31 (12)
C9—C10—H10	120.2	O6—Gd1—N3	68.37 (12)
C11—C10—H10	120.2	O7—Gd1—N3	106.25 (12)
C12—C11—C10	120.1 (5)	N4—Gd1—N3	62.59 (13)
C12—C11—C16	119.8 (4)	O3—Gd1—O14^i^	73.86 (11)
C10—C11—C16	120.1 (5)	O14^iii^—Gd1—O14^i^	77.73 (12)
C11—C12—C13	120.1 (4)	O13^i^—Gd1—O14^i^	49.79 (11)
C11—C12—H12	120.0	O4^ii^—Gd1—O14^i^	68.35 (11)
C13—C12—H12	120.0	O6—Gd1—O14^i^	158.30 (11)
O10—C13—C14	121.2 (4)	O7—Gd1—O14^i^	138.12 (11)
O10—C13—C12	118.4 (4)	N4—Gd1—O14^i^	118.82 (12)
C14—C13—C12	120.3 (5)	N3—Gd1—O14^i^	113.74 (11)
C13—C14—C9	118.9 (5)	O3—Gd1—C24^i^	86.30 (13)
C13—C14—H14	120.5	O14^iii^—Gd1—C24^i^	103.13 (13)
C9—C14—H14	120.5	O13^i^—Gd1—C24^i^	24.86 (13)
O7—C15—O6	119.9 (4)	O4^ii^—Gd1—C24^i^	69.51 (13)
O7—C15—C9	119.7 (4)	O6—Gd1—C24^i^	163.79 (12)
O6—C15—C9	120.4 (4)	O7—Gd1—C24^i^	141.23 (12)
O9—C16—O8	124.2 (5)	N4—Gd1—C24^i^	94.44 (14)
O9—C16—C11	118.1 (4)	N3—Gd1—C24^i^	95.88 (13)
O8—C16—C11	117.7 (5)	O14^i^—Gd1—C24^i^	25.87 (12)
C22—C17—C18	120.0 (5)	O3—Gd1—Gd1^ii^	68.14 (8)
C22—C17—C23	119.7 (4)	O14^iii^—Gd1—Gd1^ii^	42.54 (8)
C18—C17—C23	120.2 (4)	O13^i^—Gd1—Gd1^ii^	84.98 (9)
C17—C18—C19	119.8 (5)	O4^ii^—Gd1—Gd1^ii^	67.61 (8)
C17—C18—H18	120.1	O6—Gd1—Gd1^ii^	128.64 (8)
C19—C18—H18	120.1	O7—Gd1—Gd1^ii^	113.44 (9)
C20—C19—C18	119.7 (5)	N4—Gd1—Gd1^ii^	145.18 (10)
C20—C19—C24	120.9 (5)	N3—Gd1—Gd1^ii^	137.97 (9)
C18—C19—C24	119.3 (4)	O14^i^—Gd1—Gd1^ii^	35.19 (7)
C19—C20—C21	121.0 (5)	C24^i^—Gd1—Gd1^ii^	60.76 (10)
C19—C20—H20	119.5	O8^iv^—Gd2—O1W	76.94 (12)
C21—C20—H20	119.5	O8^iv^—Gd2—O2	144.46 (12)
O15—C21—C20	121.9 (5)	O1W—Gd2—O2	130.67 (12)
O15—C21—C22	118.8 (5)	O8^iv^—Gd2—O2W	102.62 (12)
C20—C21—C22	119.2 (5)	O1W—Gd2—O2W	147.55 (14)
C17—C22—C21	120.5 (5)	O2—Gd2—O2W	66.80 (12)
C17—C22—H22	119.8	O8^iv^—Gd2—O12	126.04 (12)
C21—C22—H22	119.8	O1W—Gd2—O12	80.37 (13)
O12—C23—O11	120.0 (4)	O2—Gd2—O12	85.07 (12)
O12—C23—C17	120.1 (4)	O2W—Gd2—O12	73.88 (12)
O11—C23—C17	119.9 (4)	O8^iv^—Gd2—O11	74.61 (12)
O13—C24—O14	120.9 (5)	O1W—Gd2—O11	74.00 (13)
O13—C24—C19	118.8 (4)	O2—Gd2—O11	129.02 (12)
O14—C24—C19	120.2 (4)	O2W—Gd2—O11	74.68 (12)
O13—C24—Gd1^i^	53.4 (2)	O12—Gd2—O11	52.15 (10)
O14—C24—Gd1^i^	71.1 (3)	O8^iv^—Gd2—O1	146.11 (12)
C19—C24—Gd1^i^	156.1 (3)	O1W—Gd2—O1	78.37 (12)
N1—C25—C26	124.6 (7)	O2—Gd2—O1	52.34 (11)
N1—C25—H25	117.7	O2W—Gd2—O1	110.66 (11)
C26—C25—H25	117.7	O12—Gd2—O1	71.28 (11)
C27—C26—C25	117.2 (8)	O11—Gd2—O1	119.78 (11)
C27—C26—H26	121.4	O8^iv^—Gd2—N1	79.62 (14)
C25—C26—H26	121.4	O1W—Gd2—N1	80.19 (16)
C26—C27—C28	120.5 (7)	O2—Gd2—N1	83.76 (14)
C26—C27—H27	119.8	O2W—Gd2—N1	132.08 (15)
C28—C27—H27	119.8	O12—Gd2—N1	142.51 (14)
C27—C28—C29	119.4 (7)	O11—Gd2—N1	146.85 (14)
C27—C28—H28	120.3	O1—Gd2—N1	73.58 (13)
C29—C28—H28	120.3	O8^iv^—Gd2—N2	67.94 (13)
N1—C29—C28	120.7 (7)	O1W—Gd2—N2	131.70 (14)
N1—C29—C30	116.9 (6)	O2—Gd2—N2	76.52 (13)
C28—C29—C30	122.4 (7)	O2W—Gd2—N2	74.37 (14)
N2—C30—C31	121.4 (7)	O12—Gd2—N2	147.56 (14)
N2—C30—C29	117.0 (6)	O11—Gd2—N2	123.70 (14)
C31—C30—C29	121.5 (7)	O1—Gd2—N2	114.77 (14)
C32—C31—C30	118.6 (8)	N1—Gd2—N2	62.11 (16)
C32—C31—H31	120.7	C25—N1—C29	117.5 (6)
C30—C31—H31	120.7	C25—N1—Gd2	119.1 (4)
C33—C32—C31	121.0 (8)	C29—N1—Gd2	123.4 (4)
C33—C32—H32	119.5	C34—N2—C30	118.2 (6)
C31—C32—H32	119.5	C34—N2—Gd2	120.9 (5)
C32—C33—C34	117.3 (8)	C30—N2—Gd2	120.6 (4)
C32—C33—H33	121.3	C35—N3—C39	117.3 (5)
C34—C33—H33	121.3	C35—N3—Gd1	123.5 (3)
N2—C34—C33	123.3 (8)	C39—N3—Gd1	119.2 (3)
N2—C34—H34	118.3	C44—N4—C40	118.2 (5)
C33—C34—H34	118.3	C44—N4—Gd1	119.9 (4)
N3—C35—C36	123.5 (6)	C40—N4—Gd1	118.9 (3)
N3—C35—H35	118.2	C7—O1—Gd2	90.8 (3)
C36—C35—H35	118.2	C7—O2—Gd2	96.0 (3)
C37—C36—C35	118.7 (6)	C8—O3—Gd1	140.6 (3)
C37—C36—H36	120.7	C8—O4—Gd1^ii^	138.4 (3)
C35—C36—H36	120.7	C3—O5—H5	109.5
C36—C37—C38	119.3 (6)	C15—O6—Gd1	94.4 (3)
C36—C37—H37	120.4	C15—O7—Gd1	93.0 (3)
C38—C37—H37	120.4	C16—O8—Gd2^iv^	138.9 (3)
C39—C38—C37	119.2 (6)	C13—O10—H10A	109.5
C39—C38—H38	120.4	C23—O11—Gd2	93.4 (3)
C37—C38—H38	120.4	C23—O12—Gd2	94.0 (3)
N3—C39—C38	122.0 (5)	C24—O13—Gd1^i^	101.7 (3)
N3—C39—C40	116.4 (4)	C24—O14—Gd1^v^	167.9 (3)
C38—C39—C40	121.4 (5)	C24—O14—Gd1^i^	83.1 (3)
N4—C40—C41	120.7 (5)	Gd1^v^—O14—Gd1^i^	102.27 (12)
N4—C40—C39	116.5 (4)	C21—O15—H15	109.5
C41—C40—C39	122.7 (5)	Gd2—O1W—H1WA	147.1
C42—C41—C40	120.4 (6)	Gd2—O1W—H1WB	105.1
C42—C41—H41	119.8	H1WA—O1W—H1WB	107.7
C40—C41—H41	119.8	Gd2—O2W—H2WA	98.9
C43—C42—C41	118.7 (6)	Gd2—O2W—H2WB	153.1
C43—C42—H42	120.6	H2WA—O2W—H2WB	107.7
C41—C42—H42	120.6	H3WA—O3W—H3WB	107.7
C42—C43—C44	118.1 (6)	H4WA—O4W—H4WB	107.7
C42—C43—H43	120.9	H5WA—O5W—H5WB	107.7
C44—C43—H43	120.9		

Symmetry codes: (i) −*x*+2, −*y*, −*z*+1; (ii) −*x*+2, −*y*+1, −*z*+1; (iii) *x*, *y*+1, *z*; (iv) −*x*+1, −*y*, −*z*; (v) *x*, *y*−1, *z*.

### Hydrogen-bond geometry (Å, °)

**Table:**

*D*—H···*A*	*D*—H	H···*A*	*D*···*A*	*D*—H···*A*
O5—H5···O12^vi^	0.82	1.92	2.720 (5)	164.
O10—H10A···O11^vii^	0.82	1.96	2.755 (5)	163.
O15—H15···O4W^v^	0.82	2.13	2.942 (10)	171.
O1W—H1WA···O3W	0.85	2.02	2.709 (6)	137.
O1W—H1WB···O9^iv^	0.85	1.90	2.622 (5)	142.
O2W—H2WA···O2	0.85	2.15	2.691 (5)	121.
O2W—H2WB···O7^viii^	0.85	1.85	2.680 (5)	165.
O3W—H3WA···O9	0.85	2.25	2.821 (7)	125.
O3W—H3WB···O4W	0.85	2.19	2.713 (7)	119.
O4W—H4WA···O6	0.85	1.97	2.813 (6)	171.
O4W—H4WB···O1	0.85	2.18	2.797 (6)	129.
O5W—H5WA···O15^iii^	0.85	1.97	2.817 (12)	173.
O5W—H5WB···O10^ix^	0.85	2.19	3.006 (13)	160.

Symmetry codes: (vi) −*x*+1, −*y*, −*z*+1; (vii) *x*+1, *y*+1, *z*; (v) *x*, *y*−1, *z*; (iv) −*x*+1, −*y*, −*z*; (viii) *x*−1, *y*−1, *z*; (iii) *x*, *y*+1, *z*; (ix) −*x*+2, −*y*+1, −*z*.
